# The effect of grape seed and green tea extracts on the pharmacokinetics of imatinib and its main metabolite, *N*-desmethyl imatinib, in rats

**DOI:** 10.1186/s40360-020-00456-9

**Published:** 2020-11-16

**Authors:** Ruba S. Darweesh, Tamam El-Elimat, Aref Zayed, Tareq N. Khamis, Wahby M. Babaresh, Tawfiq Arafat, Ahmed H. Al Sharie

**Affiliations:** 1 Department of Pharmaceutical Technology, Faculty of Pharmacy, Jordan University of Science and Technology, P.O. Box 3030, Irbid, 22110 Jordan; 2 Department of Medicinal Chemistry and Pharmacognosy, Faculty of Pharmacy, Jordan University of Science and Technology, Irbid, 22110 Jordan; 3 Jordan Center for Pharmaceutical Research (JCPR), Amman, 11195 Jordan; 4 Faculty of Medicine, Jordan University of Science and Technology, Irbid, 22110 Jordan

**Keywords:** Imatinib, *N*-desmethyl imatinib, Grape seed, Green tea, Pharmacokinetics, CYP3A, Herb-drug interactions

## Abstract

**Background:**

Imatinib is mainly metabolized by CYP3A4 and to a lesser extent by other isoenzymes, with *N*-desmethyl imatinib being its major equipotent metabolite. Being a CYP3A4 substrate, imatinib co-administration with CYP3A4 modulators would change its pharmacokinetic profile. The cancer chemoprevention potential and anticancer efficacy of many herbal products such as grape seed (GS) and green tea (GT) extracts had led to an increase in their concomitant use with anticancer agents. GS and GT extracts were demonstrated to be potent inhibitors of CYP3A4. The aim of this study is to investigate the effect of standardized GS and/or GT extracts at two different doses on the pharmacokinetics of imatinib and its metabolite, *N*-desmethyl imatinib, in SD-rats.

**Methods:**

Standardized GS and/or GT extracts were administered orally once daily for 21 days, at low (*l*) and high (*h*) doses, 50 and 100 mg/kg, respectively, before the administration of a single intragastric dose of imatinib. Plasma samples were collected and analyzed for imatinib and *N*-desmethyl imatinib concentrations using LC-MS/MS method, then their non-compartmental pharmacokinetic parameters were determined.

**Results:**

*h*-GS dose significantly decreased imatinib’s C_max_ and the ${\mathrm{AUC}}_0^{\infty }$ by 61.1 and 72.2%, respectively. Similar effects on *N*-desmethyl imatinib’s exposure were observed as well, in addition to a significant increase in its clearance by 3.7-fold. *l*-GT caused a significant decrease in imatinib’s C_max_ and ${\mathrm{AUC}}_0^{\infty }$ by 53.6 and 63.5%, respectively, with more significant effects on *N*-desmethyl imatinib’s exposure, which exhibited a significant decrease by 79.2 and 81.1%, respectively. *h*-GT showed similar effects as those of *l*-GT on the kinetics of imatinib and its metabolite. However, when these extracts were co-administered at low doses, no significant effects were shown on the pharmacokinetics of imatinib and its metabolite. Nevertheless, increasing the dose caused a significant decrease in C_max_ of *N*-desmethyl imatinib by 71.5%.

**Conclusions:**

These results demonstrated that the pharmacokinetics of imatinib and *N*-desmethyl imatinib had been significantly affected by GS and/or GT extracts, which could be partially explained by the inhibition of CYP3A-mediated metabolism. However, the involvement of other kinetic pathways such as other isoenzymes, efflux and uptake transporters could be involved and should be characterized.

**Graphical abstract:**

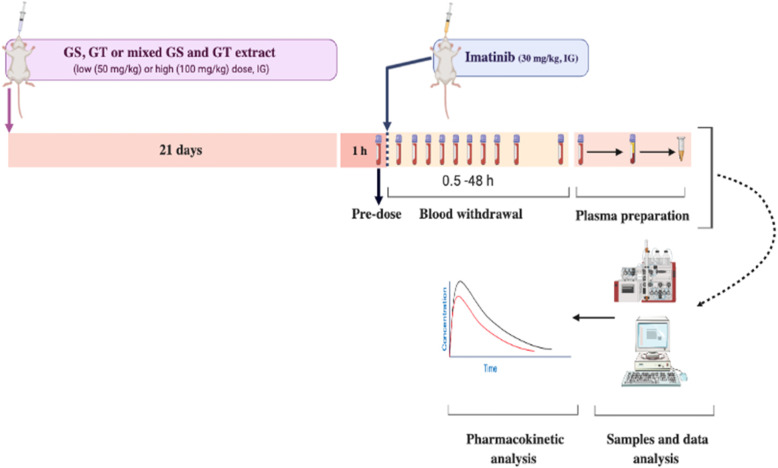

**Supplementary Information:**

The online version contains supplementary material available at 10.1186/s40360-020-00456-9.

## Background

There is a steady increase in the use of botanical/herbal products for a wide array of health problems in the last decades [1]. It has been estimated that herbal products are being used by approximately 20% of the population and the majority of those individuals use such products on a routine basis [2, 3]. Large percentage of the regular users of botanical supplements (70%) also take prescription medications. However, less than 40% of patients reveal the use of herbal dietary supplements to their physicians or other health care professionals [4]. Creating a concern of the herbal products in addition to their components altering the pharmacokinetic characteristics of the prescribed drugs, which may lead to a clinical significant interactions and adverse effects [5]. Especially since herbal products are not subjected to the same rigorous regulations of safety and efficacy required for prescription drugs approval. As a result, there is often incomplete knowledge regarding the interactions between herbal products and conventional drugs [6].

A major safety concern is the potential interactions between herbal products and anticancer drugs. The estimated worldwide prevalence of the use of herbal products by cancer patients increased from 31% in the late 1990s to 83% in the early 2000s [5]. Cancer patients use herbal products more frequently when compared to the general population to improve the quality of life and the immune system, to decrease the progression of cancer or to reduce side effects of chemotherapy [7]. However, such concomitant use and its consequent effects on the pharmacokinetics of anticancer drugs are not completely studied.

Imatinib, a tyrosine kinase inhibitor, is indicated in the treatment of chronic myeloid leukemia, gastrointestinal stromal tumors [8, 9] and other hematological and oncological diseases. It is rapidly absorbed after oral administration with high oral bioavailability (98%) with C_max_ that is achieved within 2–4 h [10]. The elimination half-life after oral administration of imatinib and its main active metabolite, *N*-desmethyl derivative are 18 and 40 h, respectively [10]. Despite its pharmacokinetic profile that favors the daily dosing, imatinib has been shown to interact with several metabolizing enzymes, which are one of the main sites of drug-drug interactions. It is mainly metabolized by CYP3A4 and to a lesser extent by CYP1A2, CYP2D6, CYP2C9 and CYP2C19 [10, 11]. Imatinib metabolism via CYP3A isoenzymes results in the formation of several metabolites, with *N*-desmethyl derivative being the most abundant one with equipotent activity to imatinib [10].

Being a CYP3A4 substrate, imatinib co-administration with CYP3A4 modulators would change the pharmacokinetic profile of imatinib [10]. For example, the combined use of imatinib with St. John’s wort, a CYP3A4 inducer, resulted in 30% reduction of the area under the curve (AUC) of imatinib [12, 13]. In addition, the combination of aprepitant, a CYP3A4 and CYP2C9 inducer, with imatinib had led to a decrease in imatinib’s bioavailability [14]. Although imatinib pharmacokinetic interactions with many conventional drugs have been studied [10], studies of the potential interactions of imatinib with the commonly used herbal products are lacking.

The cancer chemoprevention potential and anticancer efficacy of many herbal products and dietary supplements such as grape seed (GS) and green tea (GT) extracts had led to an increase in the concomitant use of these products with anticancer agents [5, 15]. Their potential combined use had raised the possible risk of pharmacokinetic interactions between these products and anticancer drugs. GS extract, which is produced from the seeds of grapes [*Vitis vinifera* L. (Vitaceae)], is rich in polyphenols that exist in their seeds as dimers, trimers and oligomers of procyanidins [16, 17]. Moreover, GS extracts are rich with procyanidins, which are reported to protect against drug- and chemical-induced multi-organ toxicity. Furthermore, it was reported that GS extracts may have cancer chemo-preventive properties against breast-, lung-, prostate-, skin- and gastro-intestinal-cancers by protecting and maintaining the growth and viability of normal cells while selectively inducing cytotoxicity toward human cancer cells [18]. On the other hand, GT extracts, which are derived from the young leaves of green tea [*Camellia sinensis* (L.) Kuntze (Theaceae)], have been known to have various pharmacological effects [19, 20], including anti-cancer [21], anti-oxidant [22], anti-obesity [23], anti-infection [24], anti-aging [25], anti-diabetic [26], and cardio-protective effects [27]. GT extracts contain characteristic polyphenols, such as (−)-epigallocatechin-3-gallate, (−)-epigallocatechin, (−)-epicatechin-3-gallate, and (−)-epicatechin in addition to different flavanols, which have been shown to play pivotal roles in the aforementioned health benefits and actions of GT extracts [20]. GS and GT extracts were demonstrated to act as potent inhibitors of CYP3A4-mediated metabolism of multiple substrates in vitro and in vivo [28–31].

The previously mentioned facts indicate that the complementary use of GS and/or GT extracts by cancer patients should be studied in terms of pharmacokinetic interactions with different anti-cancer agents. However, such pharmacokinetic studies are seriously lacking. In the light of such combination’s potential risk on the pharmacokinetic profiles of conventional chemotherapies, the current study aimed to investigate the effect of GS and/or GT extracts, potent CYP3A inhibitors, at two different doses on the pharmacokinetic parameters of imatinib, a CYP3A substrate, and its main metabolite, *N*-desmethyl imatinib, in a murine model.

## Methods

### Chemicals and reagents

Imatinib (98.0%), *N*-desmethyl-imatinib (98.0%), imatinib-d8 (98.0%), and *N*-desmethyl-imatinib-d8 (95.0%) standards were purchased from Alsachim (France). Standardized dried grape seed extract (proanthocyanidins 95% w/w) was obtained from Sanat Products Ltd., India, while standardized dried green tea extract (polyphenols ≥ 30% w/w) was obtained from Eastsign foods (Quzhou) Co. Ltd., China. Samples of grape seed and green tea extracts were stored at the Herbarium of the Faculty of Pharmacy, Jordan University of Science and Technology, Irbid, Jordan. Ketoconazole (KTZ) was a kind gift from Tabuk Pharmaceuticals, Jordan. Dimethyl sulfoxide and methanol were obtained from Scharlau Labs, Spain.

### Animals and pharmacokinetic study

The study protocol was approved by the Animal Care and Use Committee (ACUC) at Jordan University of Science and Technology (JUST), Irbid, Jordan. All study procedures were carried out in accordance with the National Institutes of Health guide for the care and use of laboratory animals. Male Sprague Dawley rats (*n* = 48; weight: 244–314 g) were obtained from the Animal Care and Breeding Facility of JUST, Irbid, Jordan. Rats were kept in clean plastic cages at room temperature of 25 ± 2 °C with a 12/12 h light/dark cycle and ~ 50% relative humidity. The experiments were carried out during the light cycle. The animals were acclimatized to laboratory conditions for a week before the beginning of the experiments, and they were provided with water and standard rat chow diet ad libitum. During housing and experiments, animals were monitored twice daily and weighed every 3 days. No adverse events were observed. Rats have fasted overnight before the beginning of the experiments with access to filtered tap water ad libitum*,* they were provided with the standard rat chow diet 2 h after imatinib administration. All animal experiments were held in the Animal Care and Breeding Facility of JUST, Irbid, Jordan.

**Fig. 1:**
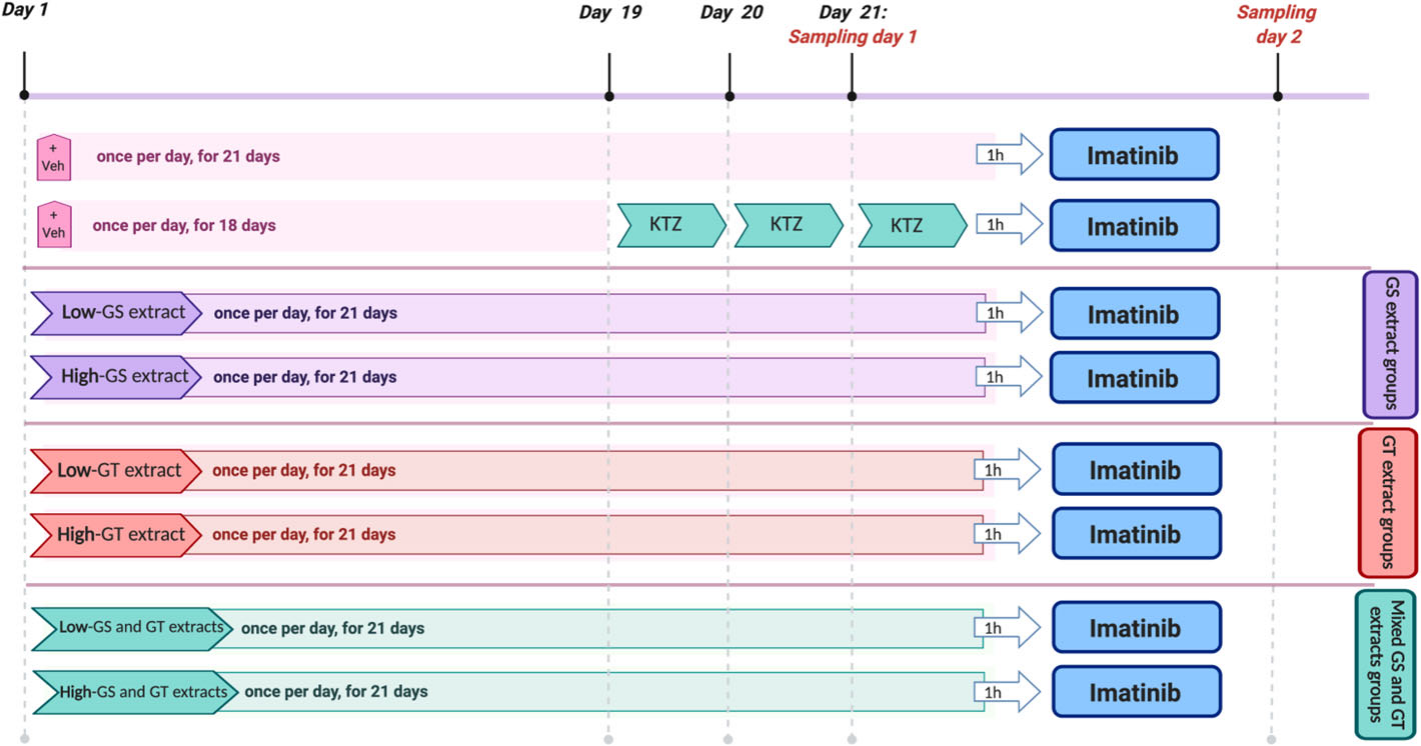
Scheme of the pharmacokinetics study design in different groups: control (imatinib only), positive control (+KTZ), grape seed extract groups (“+*low*-GS” and “+*high*-GS”), green tea extract groups (“+*low*-GT” and “+*high*-GT”, and mixed extracts groups (“+*low*-GS and GT” and “+*high*-GS and GT”). Imatinib in all groups was given as a single dose of imatinib (30 mg/kg; dissolved in DMSO) by intragastric (IG) oral gavage. KTZ was given as a single daily dose (75 mg/kg; IG, dissolved in DMSO), for three consecutive days. Low dose refers to 50 mg/kg/day, IG, of GS and/or GT extracts. High dose refers to 100 mg/kg/day, IG, of GS and/or GT extracts. Extracts were administered for 21 days before imatinib administration. Control and positive control groups were given an equivalent volume of DMSO (extracts’ vehicle, IG; “+Veh”) for 21 and 18 days, respectively

Rats were divided randomly into eight groups (6 rats per group); control, positive control, low dose of grape seed extract (*l*-GS), high dose of grape seed extract (*h*-GS), low dose of green tea extract (*l*-GT), high dose of green tea extract (*h*-GT), mixed low dose of GS and GT (*l*-GS and *l*-GT), and mixed high dose of GS and GT (*h*-GS and *h*-GT), as summarized in Fig. 1. Animals were blindly and randomly assigned to different groups by an Animal Care and Breeding Facility’s technician. Also, the experimenters were blinded to the given treatment during the treatments’ administration, samples’ analysis, data processing and evaluation.

The rats in the control group were administered a single dose of imatinib (30 mg/kg; dissolved in DMSO) by intragastric (IG) oral gavage. The positive control group was given, by IG oral gavage, a single daily dose of KTZ (75 mg/kg; dissolved in DMSO), for three consecutive days to ensure the reported CYP3A inhibition as described by Li et al. [32, 33]. On the third day, a single dose of imatinib (30 mg/kg, IG) was administered to the rats one-hour post-administration of KTZ. KTZ was not administered for 21 days to prevent the reported risks and toxicological effects caused by its long-term administration [34–38]. To confirm that any changes in the pharmacokinetics of imatinib and *N*-desmethyl-imatinib are caused only by the administered extracts (grape seed and green tea extracts) in other groups; rats in both the control and the positive control groups were given DMSO (the solvent of the extracts, 1 mL/kg) for 21 days and 18 days, respectively.

In *l*-GS and *h*-GS groups, rats received a single daily dose, by IG oral gavage, of a standardized grape seed extract (GS) of 50 and 100 mg/kg (dissolved in DMSO), respectively, for 21 days. Similarly, in *l*-GT and *h*-GT groups; rats received a single daily dose of a standardized green tea extract (GT) of 50 and 100 mg/kg (dissolved in DMSO), respectively, for 21 days. In “*l*-GS and *l*-GT” group, rats co-administered low dose of both GS and GT extracts (50 mg/kg, each), for 21 days. Similarly, rats in “*h*-GS and *h*-GT” group, co-administered high dose of both GS and GT extracts (100 mg/kg, each), for 21 days. The duration of the extract(s) administration (i.e. 21 days) was chosen as a chronic multiple-dose administration [39–41] to simulate the habitual dietary exposure [42] with its related metabolic and/or transport pathways. After 1 h of the last dose of either/both extracts (i.e. at the day of samples’ withdrawal), a single dose of imatinib (30 mg/kg; IG) was administered. After each administration of imatinib, blood samples (~ 150–200 *μ*L) were collected at the following time points: 0 (pre-dose), 0.5, 1, 2, 3, 4, 6, 8, 24, 30, and 48 h. Blood samples were collected from the tail vein without the need for any anesthesia. Plasma was collected by centrifugation at 1500×*g* for 10 min, after which the supernatant was collected into clean micro-tubes and stored at − 80 °C until analysis. All animals were executed by subtotal exsanguination under anesthesia via intraperitoneal sodium thiopental (40 mg/kg).

### Quantification of imatinib and its metabolite *N*-desmethyl imatinib

Imatinib and its metabolite, *N*-desmethyl imatinib, were quantified in plasma samples using LC-MS/MS. Briefly, plasma samples (100 *μ*L) were spiked and mixed with internal standard (IS) mixture (50 *μ*L) consisted of imatinib-d8 and *N*-desmethyl-imatinib-d8 solution. Ice-cold methanol (300 *μ*L) followed by ammonium formate solution (5 M, 50 *μ*L) were added to the samples and then mixed. Samples were centrifuged at 12000 RPM and a supernatant (~ 150 *μ*L) was transferred into HPLC vials for LC-MS/MS analysis.

### Instrumentation and chromatographic conditions

Mass spectrometric analysis was done using API 3200 triple quadrupole instrument, ABI-SCIEX (Concord, ON, Canada) equipped with 1200 series HPLC system, Agilent Technologies (Stuttgart, Germany). Data processing was done using Analyst 1.5.1 software package, SCIEX (Concord, ON, Canada). Chromatographic separation was carried out using Agilent Zorbax eclipse C_18_ column (150 mm × 5 mm, 4.6 *μ*m). The mobile phase (flow rate = 1 mL/min) consisted of 5 mM ammonium formate/methanol (80:20, v/v) (pH ~ 9.6). The analytes and their internal standards were detected in the positive ionization mode and monitored in multi-reaction monitoring (MRM) mode. The following MRM transitions *m*/*z* 494.23 → 394.10; *m*/*z* 480.34 → 394.2; *m*/*z* 502.29 → 394.20; *m*/*z* 488.29 → 394.20; were used for imatinib, *N*-desmethyl imatinib, imatinib-d8, and *N*-desmethyl imatinib-d8, respectively. The calibration curve standards ranged from 3.25 to 6662.58 ng/mL for imatinib and 3.83–980.55 ng/mL for *N*-desmethyl imatinib.

### Pharmacokinetic analysis

Non-compartmental pharmacokinetics analysis was performed using Phoenix WinNonlin 8.1 (Certara USA, Inc., USA). The maximum plasma concentration (C_max_) and time to maximum concentration (t_max_) were estimated directly from the maximum peak on plasma concentration vs. time profiles. The total area under the plasma curve (${\mathrm{AUC}}_0^{\infty }$) was calculated using the linear trapezoidal method. The mean residence time (MRT) was calculated according to ($\mathrm{MRT}=\frac{{\mathrm{AUMC}}_0^{\infty }}{{\mathrm{AUC}}_0^{\infty }}\Big)$, where ${\mathrm{AUMC}}_0^{\infty }$ is the total area under the first moment curve. The apparent volume of distribution during the terminal phase (Vz/F) was calculated using (${\mathrm{V}}_{\mathrm{z}}/\mathrm{F}=\frac{\mathrm{Dose}}{{\mathrm{AUC}}_0^{\infty}\times {\uplambda}_{\mathrm{z}}}$), where, λ_z_ is the terminal elimination rate constant, which was calculated from the plasma concentration versus time terminal slope, and *F* is the bioavailability. The apparent total clearance (Cl/F) was calculated by ($\mathrm{CL}/\mathrm{F}=\frac{\mathrm{Dose}}{{\mathrm{AUC}}_0^{\infty }}$). Finally, the terminal half-life (t_0.5_) was determined by (${\mathrm{t}}_{0.5}=\frac{0.693}{\uplambda_{\mathrm{z}}}\Big)$.

### Statistical analysis

Data was reported as Mean ± SD. JMP 14.3 software (SAS Institute, NC, USA), was used for all statistical analyses with a significance level of 0.05. Student *t*-test and analysis of variance (ANOVA) were used for two-or multiple-group comparisons, respectively, and in the event of multiple comparisons; Tukey’s multi-comparison test was used.

## Results

### Pharmacokinetics of imatinib and its metabolite, *N*-desmethyl imatinib

**Fig. 2:**
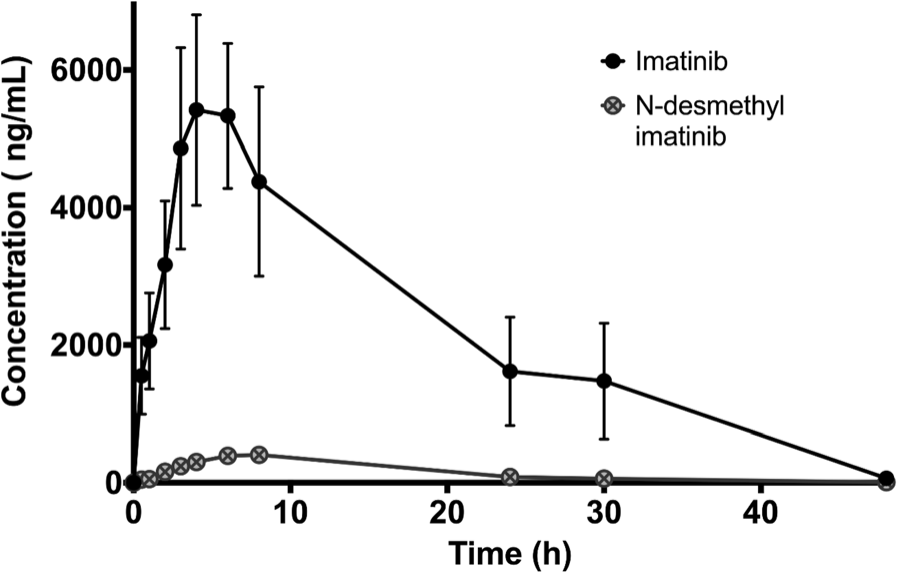
Plasma concentration of imatinib in closed black circle and its metabolite, *N*-desmethyl imatinib, in open-crossed grey circles vs. time profiles in the control (imatinib only) group. Data: mean ± SE (*n* = 5–6)

The plasma concentration versus time profiles of imatinib and its metabolite *N*-desmethyl imatinib after oral administration of imatinib are shown in Fig. 2. The pharmacokinetic parameters of imatinib and *N*-desmethyl imatinib are summarized in Tables 1 and 2, respectively. Imatinib’s C_max_ and ${\mathrm{AUC}}_0^{\infty }$ were shown to be 6399.6 ± 3162.8 ng/mL and 105,160.8 ± 75,643.2 ng^.^h/mL, respectively. Imatinib has t_max_ and a terminal half-life of 4.8 ± 2.3 h and 6.2 ± 0.8 h, respectively. On the other hand, *N*-desmethyl-imatinib has C_max_ and ${\mathrm{AUC}}_0^{\infty }$ of 465.6 ± 94.0 ng/mL and 7143.3 ± 2165.3 ng^.^h/mL, t_max_ and terminal half-life of 6.7 ± 2.1 and 6.6 ± 0.5, respectively. C_max (metabolite)_ /C_max (drug)_ and ${\mathrm{AUC}}_{0\ \left(\mathrm{metabolite}\right)}^{\infty }/{\mathrm{AUC}}_{0\ \left(\mathrm{drug}\right)}^{\infty }$ percentages were found to be 7.3 and 6.8, respectively. Tables S1 and S2 (supplementary data) include statistical differences, presented as percentages of decrease or folds of increase, in pharmacokinetic parameters of imatinib and *N*-desmethyl imatinib among different study groups, respectively.

**Table 1:** Pharmacokinetic parameters of **imatinib** in different groups

	Parameter (unit)						
	C_**max**_ (ng/mL)	t_**max**_ (h)	t_**0.5**_ (h)	${\mathbf{AUC}}_{\mathbf{0}}^{\mathbf{\infty}}$ **(ng**^**.**^**h/mL)**	MRT (h)	V_**z**_/F (mL/kg)	CL/F (mL/h/kg)
**Imatinib only**	6399.6 ± 3162.8	4.8 ± 2.3	6.2 ± 0.8	105,160.8 ± 75,643.2	11.1 ± 4.8	6.1 × 10^− 3^ ± 7.8 × 10^− 3^	0.6 × 10^− 3^ ± 0.7 × 10^− 3^
**Imatinib + KTZ**	2320.5* ± 448.8	13.5 ± 11.0	5.9 ± 0.8	52,747.1 ± 9652.7	17.5 ± 5.4	5.0 × 10^− 3^ ± 1.4 × 10^− 3^	0.6 × 10^− 3^ ± 0.1 × 10^− 3^
**Imatinib +** ***l*-GS**	5153.2 ± 1902.4	3.7 ± 0.5	5.7 ± 0.2	59,233.8 ± 15,601.1	7.4 ± 0.5	4.4 × 10^− 3^ ± 1.3 × 10^− 3^	0.5 × 10^− 3^ ± 0.2 × 10^− 3^
**Imatinib +** ***h*-GS**	2487.3* ± 1141.8	4.8 ± 1.8	5.2* ± 0.6	29,229.5* ± 10,979.2	7.7 ± 1.0	9.0 × 10^− 3^ ± 4.4 × 10^− 3^	1.2 × 10^− 3^ ± 0.4 × 10^− 3^
**Imatinib +** ***l*-GT**	2972.3* ± 1686.6	5.5 ± 2.2	5.0* ± 0.3	38,387.3* ± 17,570.8	9.8 ± 2.8	6.5 × 10^− 3^ ± 2.5 × 10^− 3^	0.9 × 10^− 3^ ± 0.4 × 10^− 3^
**Imatinib +** ***h*-GT**	3225.6* ± 611.7	4.5 ± 2.2	5.2* ± 0.6	42,764.2 ± 6215.1	8.5 ± 1.6	5.4 × 10^−3^ ± 1.1 × 10^− 3^	0.7 × 10^− 3^ ± 0.1 × 10^− 3^
**Imatinib +** ***l*-GS and** ***l*-GT**	9403.7^**†‡**^ ± 1500.3	4.7 ± 2.0	6.3^**†‡**^ ± 0.3	151,343.8^**†‡**^ ± 27,244.5	12.8^**†**^ ± 2.9	1.8 × 10^–3**†**^ ± 0.3 × 10^− 3^	0.2 × 10^− 3^ ± 0.0
**Imatinib +** ***h*-GS and** ***h*-GT**	3711.8^#^ ± 2546.6	5.0 ± 2.7	4.8*^#^ ± 0.7	50,340.5^#^ ± 34,035.6	7.7^#^ ± 0.6	8.2 × 10^−3^ ± 7.6 × 10^− 3^	1.3 × 10^− 3^ ± 1.3 × 10^− 3^

Pharmacokinetic parameters of **imatinib** after imatinib (30 mg/kg) administration alone (control), with ketoconazole (KTZ; 75 mg/kg), with a single low dose of grape seed (GS) or green tea (GT) extracts (*l*-GS or *l*-GT; 50 mg/kg), with a single high dose of GS or GT extracts (*l*-GS or *l*-GT; 100 mg/kg), or with a single co-administered low dose of both GS and GT extracts, or with a single co-administered high dose of both GS and GT extracts. Data: mean ± SD (*n* = 5–6)

^*^p-value < 0.05, compared to control (i.e. imatinib only)

^#^*p*-value < 0.05, when compared to the *l*ow dose of each designated group

^**†**^*p*-value < 0.05, when “imatinib+ *l*-GS and *l*-GT” group is compared to *l*-GS group

^**‡**^*p*-value < 0.05, when “Imatinib+ *l*-GS and *l*-GT” group is compared to *l*-GT group

**Table 2:** Pharmacokinetic parameters of ***N*-desmethyl imatinib** in different groups

	Parameter (unit)						
	C_**max**_ (ng/mL)	t_**max**_ (h)	t_**0.5**_ (h)	$$ {\mathbf{AUC}}_{\mathbf{0}}^{\mathbf{\infty}} $$ **(ng**^**.**^**h/mL)**	MRT (h)	V_**z**_/F (mL/kg)	CL/F (mL/h/kg)
**Imatinib only**	465.6 ± 94.0	6.7 ± 2.1	6.6 ± 0.5	7143.3 ± 2165.3	11.6 ± 3.8	4.31 × 10^− 3^ ± 13.8 × 10^− 3^	4.5 × 10^− 3^ ± 1.4 × 10^− 3^
**Imatinib + KTZ**	79.9* ± 15.8	7.5 ± 1.0	9.9 ± 4.4	1724.8* ± 690.7	18.0* ± 3.6	0.3 ± 0.2	19.2 × 10^− 3^* ± 6.1 × 10^− 3^
**Imatinib +** ***l*-GS**	408.8 ± 113.6	4.2 ± 1.0	6.2 ± 0.5	4638.9* ± 825.6	8.0* ± 0.9	60.1 ± 13.9 × 10^− 3^	6.6 × 10^− 3^ ± 1.2 × 10^− 3^
**Imatinib +** ***h*-GS**	149.8*^#^ ± 45.3	4.7 ± 2.0	7.1 ± 0.9	1926.6*^#^ ± 526.0	9.5 ± 1.0	0.2*^#^ ± 0.1	16.7 × 10^− 3^*^#^ ± 5.0 × 10^− 3^
**Imatinib +** ***l*-GT**	96.4* ± 41.1	5.3 ± 1.6	8.0* ± 0.7	1351.8* ± 455.7	11.2 ± 1.8	0.3* ± 0.1	25.0 × 10^−3^* ± 10.5 × 10^− 3^
**Imatinib +** ***h*-GT**	170.8* ± 119.8	5.2 ± 2.6	7.6* ± 0.5	2547.9* ± 1905.0	11.9 ± 2.6	0.2 ± 0.1	19.5 × 10^−3^* ± 13.4 × 10^− 3^
**Imatinib +** ***l*-GS and** ***l*-GT**	472.7^**‡**^ ± 72.2	5.8 ± 2.6	8.1^**†**^ ± 0.6	8252.7^**†‡**^ ± 1207.8	12.8^**†**^ ± 2.3	42.6 × 10^−3^ ± 4.2 × 10^− 3^	3.7 × 10^− 3^ ± 0.5 × 10^− 3^
**Imatinib +** ***h*-GS and** ***h*-GT**	132.9*^#^ ± 95.3	4.8 ± 2.2	10.3*^#$§^ ± 1.8	1995.6*^#^ ± 1326.6	11.9 ± 2.3	0.4*^#^ ± 0.3	24.0 × 10^−3^*^#^ ± 18.4 × 10^− 3^

Pharmacokinetic parameters of ***N*-desmethyl imatinib** after imatinib (30 mg/kg) administration alone (control), with ketoconazole (KTZ; 75 mg/kg), with a single low dose of grape seed (GS) or green tea (GT) extracts (*l*-GS or *l*-GT; 50 mg/kg), with a single high dose of GS or GT extracts (*l*-GS or *l*-GT; 100 mg/kg), or with a single co-administered low dose of both GS and GT extracts, or with a single co-administered high dose of both GS and GT extracts. Data: mean ± SD (*n* = 4–6)

^*^p-value < 0.05, compared to control (i.e. imatinib only)

^#^*p*-value < 0.05, when compared to *l*ow dose of each designated group

^**†**^*p*-value < 0.05, when “imatinib+ *l*-GS and *l-*GT” group is compared to *l*-GS group

^**‡**^*p*-value < 0.05, when “imatinib+ *l*-GS and *l-*GT” group is compared to *l*-GT group

^$^*p*-value < 0.05, when “imatinib+ *h*-GS and *h*-GT” group is compared to *h*-GS group

^§^*p*-value < 0.05, when “imatinib+ *h*-GS and *h*-GT” group is compared to *h*-GT group

**Fig. 3:**
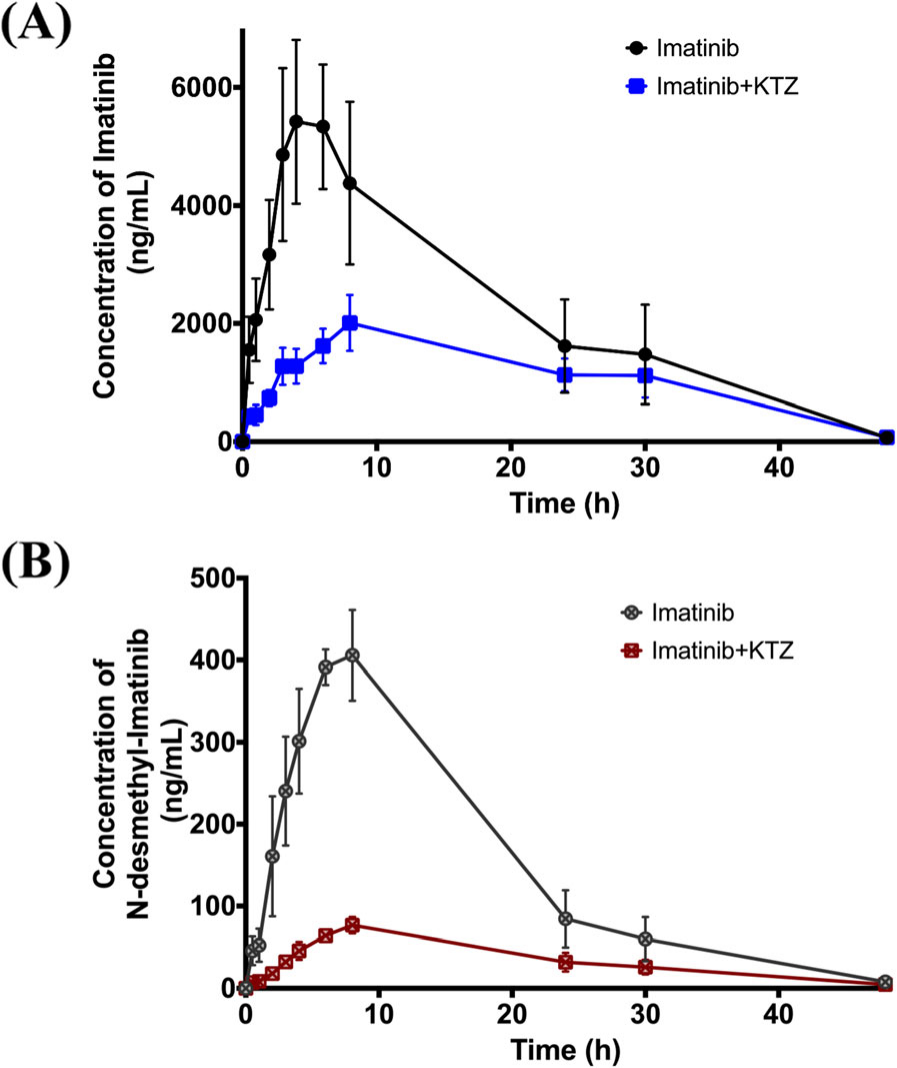
Plasma concentration vs. time profiles of (**a**) imatinib; in the control (imatinib only) group and the positive control group (imatinib and KTZ) in closed circles, and squares, respectively. **b** *N*-desmethyl imatinib; in the control (imatinib only) group and the positive control group (imatinib and KTZ) in open-crossed circles, and squares, respectively. Data: mean ± SE (*n* = 5–6). KTZ: ketoconazole

When ketoconazole (KTZ) was co-administered with imatinib, it caused a significant decrease in C_max_ of imatinib by 63.7% (*p*-value < 0.05), compared to the control group, i.e. imatinib only (Table 1). This effect was accompanied by a significant decrease in C_max_ and ${\mathrm{AUC}}_0^{\infty }$ of *N*-desmethyl imatinib by 82.8 and 75.9% (*p*-value < 0.05), respectively. In addition to a significant increase in MRT and apparent clearance of *N*-desmethyl imatinib by 1.6- and 4.3-fold, respectively (*p*-value < 0.05), when compared to the control group (Table 2). Percentages of C_max (metabolite)_ /C_max (drug)_ and ${\mathrm{AUC}}_{0\ \left(\mathrm{metabolite}\right)}^{\infty }/{\mathrm{AUC}}_{0\ \left(\mathrm{drug}\right)}^{\infty }$ were 3.4 and 3.3, respectively. The plasma concentration versus time profiles of imatinib and its metabolite, *N*-desmethyl imatinib, after co-administration of imatinib and KTZ are shown in Fig. 3a and b, respectively.

### Grape Seed (GS) extract decreases the systemic exposure of imatinib and its metabolite, and increases the clearance of *N*-desmethyl imatinib

**Fig. 4:**
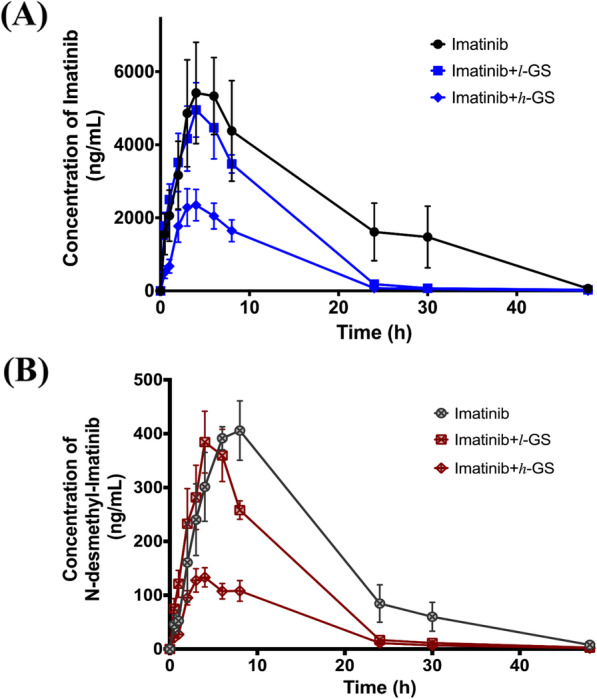
Plasma concentration vs. time profiles of (**a**) imatinib; in the control (imatinib only) group, imatinib+ *l*-GS group, and imatinib+ *h*-GS group, in closed circles, squares, and diamonds, respectively. **b** *N*-desmethyl imatinib; in the control (imatinib only) group, imatinib+ *l*-GS group, and imatinib+ *h*-GS group, in open-crossed circles, squares, and diamonds, respectively. Data: mean ± SE (*n* = 5–6). *l*-GS and *h*-GS: low and high dose of grape seed extract, respectively

The pharmacokinetic parameters of imatinib and *N*-desmethyl imatinib after imatinib co-administration with low (50 mg/kg) or high (100 mg/kg) dose of grape seed extract (*l*-GS and *h*-GS) are summarized in Tables 1 and 2, respectively. When low dose of GS was administered for 3 weeks before the imatinib dose, no significant differences in imatinib pharmacokinetic parameters were observed, when compared to the control group (Fig. 4a, Table 1). However, *N*-desmethyl imatinib ${\mathrm{AUC}}_0^{\infty }$ and MRT significantly decreased by 35.1 and 31.2%, respectively (*p*-value < 0.05), when compared to the control group (Fig. 4b, Table 2). C_max (metabolite)_ /C_max (drug)_ and ${\mathrm{AUC}}_{0\ \left(\mathrm{metabolite}\right)}^{\infty }/{\mathrm{AUC}}_{0\ \left(\mathrm{drug}\right)}^{\infty }$ percentages were found to be of 7.9 and 7.8, respectively. When the dose of GS was doubled to 100 mg/kg in *h*-GS group, a significant decrease was observed in the C_max_ and the ${\mathrm{AUC}}_0^{\infty }$ of imatinib by 61.1 and 72.2% (*p*-value < 0.05), respectively, with also a 16.1% significant decrease in imatinib’s terminal t_0.5_ (*p*-value < 0.05), when compared to the control group (Fig. 4a). Similar effects were observed for C_max_ and ${\mathrm{AUC}}_0^{\infty }$ of *N*-desmethyl imatinib, where a significant decrease of 67.8 and 73.0% (*p*-value < 0.05), respectively, was shown in *h*-GS group (Fig. 4b). Furthermore, the apparent volume of distribution and the apparent clearance of *N*-desmethyl imatinib were significantly increased by 4.1- and 3.7-fold (*p*-value < 0.05), respectively, when compared to the control group. C_max (metabolite)_ /C_max (drug)_ and ${\mathrm{AUC}}_{0\ \left(\mathrm{metabolite}\right)}^{\infty }/{\mathrm{AUC}}_{0\ \left(\mathrm{drug}\right)}^{\infty }$ percentages were changed to be of 6.0 and 6.6, respectively.

When imatinib’s pharmacokinetic parameters of *h*-GS group were compared to those of *l*-GS group, it was shown that increasing the dose of GS had no significant effects on imatinib’s kinetics. Nevertheless, doubling the dose of GS caused a significant decrease in C_max_ and ${\mathrm{AUC}}_0^{\infty }$ of *N*-desmethyl imatinib by 63.3 and 58.5% (*p*-value < 0.05), respectively, and a significant increase in its apparent volume of distribution and apparent clearance by 2.9- and 2.5-fold (*p*-value < 0.05), respectively (Fig. 4, Tables 1 and 2).

### Green Tea (GT) extract decreases the systemic exposure and increases the clearance of both imatinib and its metabolite, *N*-desmethyl imatinib

**Fig. 5:**
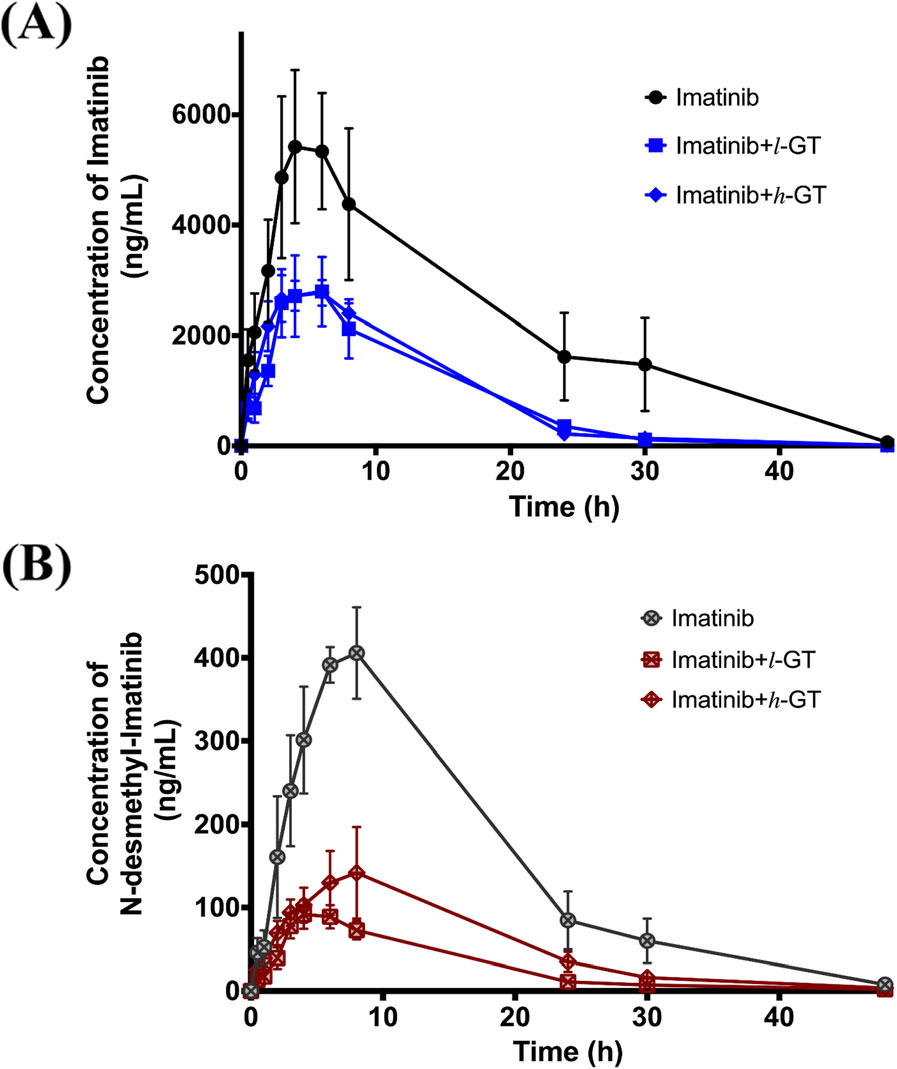
Plasma concentration vs. time profiles of (**a**) imatinib; in the control (imatinib only) group, imatinib+ *l*-GT group, and imatinib+ *h*-GT group, in closed circles, squares, and diamonds, respectively. **b** *N*-desmethyl imatinib; in the control (imatinib only) group, imatinib+ *l*-GT group, and imatinib+ *h*-GT group, in open-crossed circles, squares, and diamonds, respectively. Data: mean ± SE (*n* = 5–6). *l*-GT and *h*-GT: low and high dose of green tea extract, respectively

The pharmacokinetic parameters of imatinib and *N*-desmethyl imatinib after imatinib co-administration with low (50 mg/kg) or high (100 mg/kg) dose of green tea extract (*l*-GT and *h*-GT) are summarized in Tables 1 and 2, respectively. Imatinib pharmacokinetic parameters after 21 days of *l*-GT administration exhibited a significant decrease in C_max_, ${\mathrm{AUC}}_0^{\infty }$, and terminal t_0.5_ by 53.6, 63.5, and 19.6%, respectively (*p*-value < 0.05), when compared to the control group (Fig. 5a). More significant effects were observed on C_max_ and ${\mathrm{AUC}}_0^{\infty }$ of *N*-desmethyl imatinib, as *l*-GT caused a significant decrease by 79.2 and 81.1% (*p*-value < 0.05), respectively. Furthermore, the terminal elimination rate constant of *N*-desmethyl imatinib was decreased significantly, which caused a significant increase in its terminal t_0.5_ from 6.6 ± 0.5 h to 8.0 ± 0.7 h (*p*-value < 0.05). Significant increase in both the apparent volume of distribution and the apparent clearance of *N*-desmethyl imatinib by 6.8- and 5.6-fold (*p*-value < 0.05), respectively, were also shown when compared to the control group (Fig. 5b). This was accompanied with C_max (metabolite)_ /C_max (drug)_ and ${\mathrm{AUC}}_{0\ \left(\mathrm{metabolite}\right)}^{\infty }/{\mathrm{AUC}}_{0\ \left(\mathrm{drug}\right)}^{\infty }$ percentages of 3.2 and 3.5, respectively.

Doubling the dose of GT to 100 mg/kg, *h*-GT group, caused a significant decrease in C_max_, and terminal t_0.5_ by 49.6 and 15.8%, respectively (*p*-value < 0.05), when compared to the control group (Fig. 5a). For *N*-desmethyl imatinib, *h*-GT treatment caused C_max_ and ${\mathrm{AUC}}_0^{\infty }$ to be decreased significantly by 63.3 and 64.3% (*p*-value < 0.05), respectively. Apparent clearance and terminal t_0.5_ of *N*-desmethyl imatinib were significantly increased by 4.3- and 1.2-fold (*p*-value < 0.05), respectively, when compared to the control group (Fig. 5b). This was shown with C_max (metabolite)_ /C_max (drug)_ and ${\mathrm{AUC}}_{0\ \left(\mathrm{metabolite}\right)}^{\infty }/{\mathrm{AUC}}_{0\ \left(\mathrm{drug}\right)}^{\infty }$ percentages of 5.3 and 6.0, respectively.

Even though *l*-GT apparently affected the kinetic parameters more than *h*-GT treatment, especially for *N*-desmethyl imatinib, but comparing both *l*-GT and *h*-GT pharmacokinetic parameters for both imatinib and *N*-desmethyl imatinib showed no significant differences (*p*-value > 0.05).

### Co-administration of GS and GT extracts decreases the systemic exposure *N*-desmethyl imatinib and increases its clearance

**Fig. 6:**
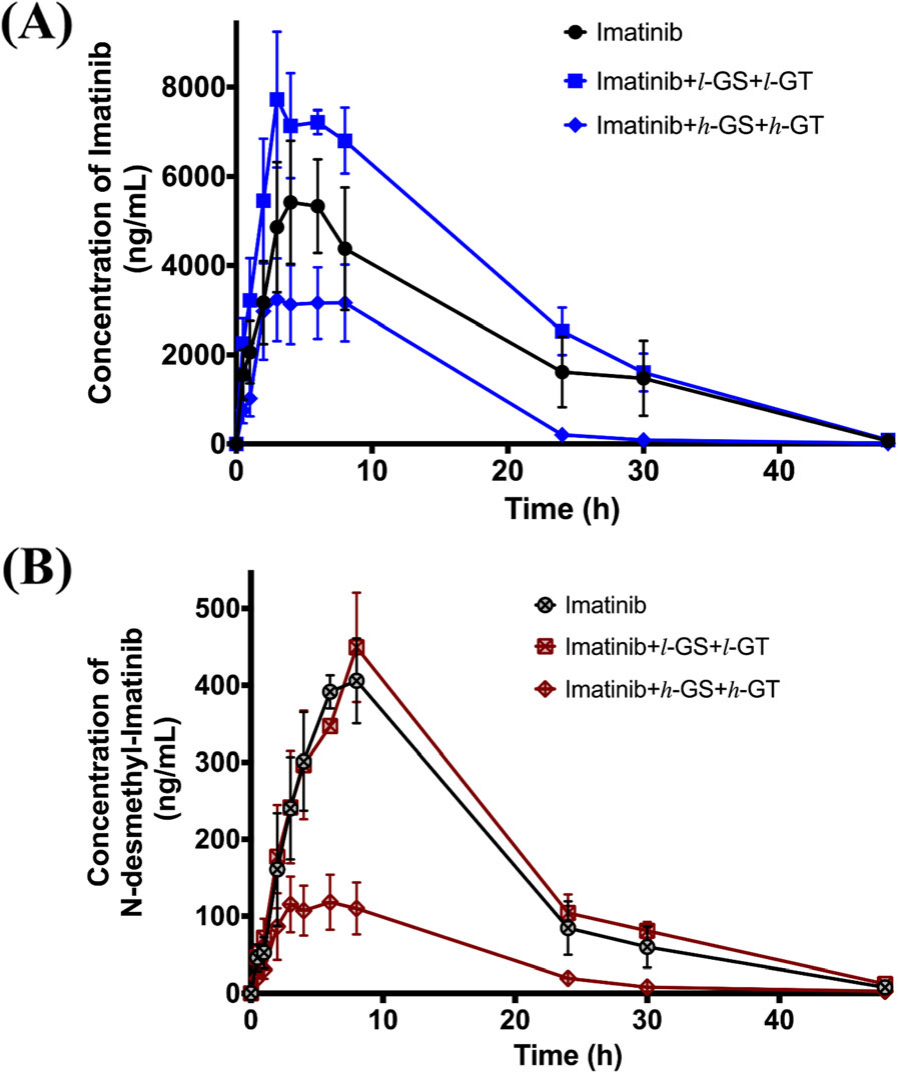
Plasma concentration vs. time profiles of (**a**) imatinib; in the control (imatinib only) group, “imatinib+ *l*-GS+ *l*-GT” group, and “imatinib+ *h*-GS+ *h*-GT” group, in closed circles, squares, and diamonds, respectively. **b** *N*-desmethyl-imatinib; in the negative control group (imatinib only), “imatinib+ *l*-GS+ *l*-GT” group, and “imatinib+ *h*-GS+ *h*-GT” group, in open-crossed circles, squares, and diamonds, respectively. Data: mean ± SE (*n* = 4–6). *l*-GS and *h*-GS: low and high dose of grape seed extract, respectively. *l*-GT and *h*-GT: low and high dose of green tea extract, respectively

The effect of co-administration of GS and GT together with imatinib was also characterized at low dose (*l*-GS and *l*-GT; 50 mg/kg each) and high dose (*h*-GS and *h*-GT; 100 mg/kg each). The pharmacokinetic parameters of imatinib and *N*-desmethyl imatinib of these groups are summarized in Tables 1 and 2. In the “*l*-GS and *l*-GT” group, the pharmacokinetics of imatinib and *N*-desmethyl imatinib were not significantly affected, when compared to the control group (*p*-value > 0.05), as shown in Fig. 6. The ratio percentages of C_max (metabolite)_ /C_max (drug)_ and ${\mathrm{AUC}}_{0\ \left(\mathrm{metabolite}\right)}^{\infty }/{\mathrm{AUC}}_{0\ \left(\mathrm{drug}\right)}^{\infty }$ were 5.0 and 5.5, respectively. However, when the dose of each administered extracts was doubled, in “*h*-GS and *h*-GT” group, the terminal t_0.5_ significantly decreased by 21.9%, when compared to the control (*p*-value < 0.05). Furthermore, co-administration of high dose of both extracts resulted in a significant decrease in the C_max_ and ${\mathrm{AUC}}_0^{\infty }$ of *N*-desmethyl imatinib by 71.5 and 72.1% (*p*-value < 0.05), respectively, and a significant increase in the terminal t_0.5_, apparent volume of distribution, and clearance by 1.6-, 8.7- and 5.3-fold (*p*-value < 0.05), respectively, when compared to the control kinetics (Fig. 6). The ratio percentages of C_max (metabolite)_ /C_max (drug)_ and ${\mathrm{AUC}}_{0\ \left(\mathrm{metabolite}\right)}^{\infty }/{\mathrm{AUC}}_{0\ \left(\mathrm{drug}\right)}^{\infty }$ were 3.6 and 4.0, respectively.

When the pharmacokinetic parameters of high dose mixed extracts group, “*h*-GS and *h*-GT”, were compared to that in low dose mixed extracts group, “*l*-GS and *l*-GT”, it was shown that imatinib’s C_max_ and ${\mathrm{AUC}}_0^{\infty }$ were significantly decreased by 60.5 and 66.7%, respectively (*p*-value < 0.05). In addition, imatinib’s terminal t_0.5_ and MRT were significantly decreased by 23.1 and 39.8%, respectively (*p*-value < 0.05). On the other hand, *N*-desmethyl imatinib’s C_max_ and ${\mathrm{AUC}}_0^{\infty }$ significantly decreased by 71.9 and 75.8% (*p*-value < 0.05), respectively, while its terminal t_0.5_, apparent volume of distribution and clearance were significantly increased by 1.3-, 8.8- and 6.5-fold (*p*-value < 0.05), respectively.

### Co-administration of GS and GT extracts has less effect in decreasing imatinib’s systemic exposure and increasing its clearance, when compared to the administration of each extract separately

The effect of low dose administration of each extract versus the co-administration of both extracts at low dose were compared. It was shown that co-administration of “*l*-GS and *l*-GT” with imatinib, when compared to *l*-GS group, caused a significant increase in C_max_ and ${\mathrm{AUC}}_0^{\infty }$ of imatinib by 1.8- and 2.6-fold, and a significant increase in terminal t_0.5_ and MRT, by 1.1- and 1.7-fold (*p*-value < 0.05), respectively. Imatinib’s apparent volume of distribution also significantly decreased by 59.1% (*p*-value < 0.05). Similar effects were also obtained when comparing the mixed “*l*-GS and *l*-GT” group’s kinetics to that of *l*-GT group. A significant increase in C_max_ and ${\mathrm{AUC}}_0^{\infty }$ of imatinib by 3.2- and 3.9-fold, and significant 1.3-fold increase in terminal t_0.5_ of imatinib (*p*-value < 0.05) were observed. The effects of co-administration of “*l*-GS and *l*-GT” with imatinib on *N*-desmethyl imatinib were also characterized against *l*-GS group. A significant increase in its ${\mathrm{AUC}}_0^{\infty }$, terminal t_0.5_ and MRT by 1.8-, 1.3- and 1.6-fold (*p*-value < 0.05), respectively, were observed. While when it was compared to *l*-GT’s kinetics, co-administration of *l*-GS and *l*-GT extracts caused a significant increase in C_max_ and ${\mathrm{AUC}}_0^{\infty }$ of *N*-desmethyl imatinib by 4.9- and 6.1-fold (*p*-value < 0.05), respectively (Table 2).

The kinetics of the co-administration of “*h*-GS and *h*-GT” with imatinib against other high dose groups (100 mg/kg) were also characterized. It was found that no significant effects of such co-administration on imatinib’s kinetics, when compared to either *h*-GS or *h*-GT groups’ kinetics separately, were observed (Table 1). But this co-administration caused a significant increase in the terminal half-life of imatinib and *N*-desmethyl imatinib by 1.5- and 1.4-fold (*p*-value < 0.05), respectively.

## Discussion

There is a serious concern of potential interactions between herbal products/extracts and anti-cancer agents [43]. This concern is attributed mainly to a worldwide increase in herbal products usage among cancer patients [5]. Such increased self-prescription resulted from many reports and media over promotion of the cancer chemoprevention potential and anticancer effects of many herbal products and dietary supplements [15]. Among such products are GS and GT extracts.

Imatinib mesylate is an anti-cancer agent that is sold by Novartis under the brand name Gleevec [11]. It is majorly metabolized by CYP3A4 [29] to its main metabolite, *N*-desmethyl imatinib [10, 44]. As imatinib is a CYP3A4 substrate, co-administration with CYP3A4 modulators would change its pharmacokinetic profile [10]. GS and GT extracts were reported to be potent inhibitors of CYP3A4 [28–30, 45]. Although there is a potential co-administration of imatinib and GS and/or GT extracts and therefore interaction, but no pharmacokinetic studies were found in the literature to characterize such possible interaction. Accordingly, in this study, we chose to characterize the potential interactions between imatinib and its major metabolite, *N*-desmethyl imatinib, with GS and/or GT extracts in vivo in rats.

In the current study, the dose of imatinib was chosen according to previous studies [14, 46–51]. On the other hand, the GS extracts doses in human are highly variable, which reach 2000 mg/day [52]. The human doses of GT extracts are even more variable, which would reach 4.9 g (3 g/m^2^) [53]. Therefore, the dose of GS extract in high dose groups (i.e. 100 mg/kg) was selected as described earlier [18, 40, 42, 54–56], and its half in the GS extract low dose groups. However, the GT extract dose that was reported in literature is highly variable in animal studies [57], hence, the choice of its dose in our study was to be comparable with GS extract groups of 100 mg/kg in high dose groups [58] and 50 mg/kg in low dose groups [59].

Co-administration of KTZ with imatinib was characterized earlier in vivo [46, 60]. Soo et al. and Lin et al. showed that KTZ, at a dose of 50 and 30 mg/kg, respectively, co-administration with imatinib caused an increase in imatinib’s ${\mathrm{AUC}}_0^{\infty }$ and C_max_ [46, 60]. However, KTZ did not change liver and kidney distribution and also did not affect brain penetration, most probably because of the involvement of different transporters in imatinib’s transport that would mask the inhibition of P-gp by KTZ [60]. In addition, the used dose of KTZ might be effective for first-pass metabolism inhibition, but not high enough to affect blood-brain barrier penetration [60]. In our study, the used KTZ dose was higher than those two studies implying that the KTZ dose might be high enough to inhibit P-gp efflux in tissues, therefore decreasing imatinib’s systemic exposure. Actually, the IC_50_ of CYP3A inhibition by KTZ ranged from 0.03–0.3 *μ*M [61], and of P-gp inhibition ranged from 1.2–3.4 *μ*M in vitro [62] against different CYP3A and P-gp substrates, respectively. Moreover, Lin et al. showed that KTZ (30 mg/kg) affected the pharmacokinetics of *N*-desmethyl imatinib by decreasing its ${\mathrm{AUC}}_0^{\infty }$ and C_max_, which was accompanied with an increase in its MRT and Cl/F [46]. These results are similar to our results, except that these effects were stronger in our study when compared to Lin et al. study, which might be a result of the higher KTZ dose used in our study.

In our study, the administration of low dose of GS extract (50 mg/kg) with imatinib caused a significant decrease in the extent formation/appearance of *N*-desmethyl imatinib in the systemic circulation with no significant effect on imatinib’s pharmacokinetics. This would follow the hypothesis that GS extract inhibited the metabolism of imatinib through the inhibition of its main metabolism pathway (i.e. CYP3A), which is consistent with previous reports [29, 63–65].

When the dose of GS extract was doubled to 100 mg/kg in *h*-GS group, both the C_max_ and ${\mathrm{AUC}}_0^{\infty }$ of *N*-desmethyl imatinib were significantly decreased by ~ 2-fold, indicating that both the rate and the extent of the metabolite’s exposure was decreased in a dose-dependent manner, which again could be explained by the inhibition of CYP3A metabolism pathway. However, this is expected to cause an increase in imatinib bioavailability, which was not the case in our study. The bioavailability of imatinib was significantly decreased. This is indicative that pharmacokinetic pathways, other than the CYP3A metabolism pathway, are affected by GS extract administration. This could be caused by the involvement of other metabolism and/or pharmacokinetic pathways of imatinib.

In the case of GT extract, at low dose (50 mg/kg), the effect on the metabolite was shown to be more pronounced, when compared to similar dose of GS extract, on the extent and rate of *N*-desmethyl imatinib formation and/or appearance in the systemic circulation. Interestingly, similar effects on *N*-desmethyl imatinib bioavailability were shown, but to a lesser extent when the dose of GT extract was increased. These changes would again be explained by the inhibition of CYP3A metabolism pathway as reported for GT extract [65] or its components such as (−)-epigallocatechin-3-gallate (EGCG) and (−)-epicatechin-3-gallate (ECG) [66, 67]. However, the exposure of imatinib was not increased at both doses of GT extract, which indicates that its effect involves other pharmacokinetic pathways.

Our findings in all cases emphasize that the metabolism of imatinib to *N*-desmethyl imatinib is not the only determinant of imatinib’s pharmacokinetics. Thus, the ratio of *N*-desmethyl imatinib/imatinib concentrations or total area under the curve ratios are not the key influence of imatinib’s pharmacokinetics. This was recently reported by Skoglund et al., where they showed that the activity of CYP3A4 is not necessarily the main determinant of the plasma concentration of imatinib and *N*-desmethyl imatinib, and therefore it is not the rate-limiting step in imatinib’s pharmacokinetics [68].

Although imatinib is mainly metabolized by CYP3A4 isoenzyme [69], but other isoenzymes are also involved. It was indicated that CYP2C8 plays a key role in imatinib metabolism [70], especially in the case of CYP3A4 auto-inhibition at imatinib steady state [69, 71]. Moreover, imatinib metabolism involves CYP1A1 and CYP1B1 [72] with a minor role of CYP2C9, 2D6, 2C19, and 1A2 isoenzymes [10, 73]. Uptake transporters such as the organic cation transporter 1 (OCT-1), were also found to affect the pharmacokinetics of imatinib [74, 75]. Furthermore, imatinib uptake was reported to be mediated by organic anion transporting polypeptide 1A2 (OATP1A2) and it was shown that it is a substrate for OATP1B3 and OCTN2 transporters [76, 77]. On the other hand, efflux ATP-binding cassette transporters (ABC), such as ABCB1 (P-gp) and ABCG2 (BCRP) transporters, are also involved in the pharmacokinetics of imatinib [78, 79]. Actually, *N*-desmethyl imatinib was found to be a better substrate for P-gp and BCRP transporters, when compared to imatinib [80–83]. These facts imply that the pharmacokinetics of imatinib and *N*-desmethyl imatinib are a complex of various pathways, which in the case of drug, herbal, and/or food co-administration indicates that multiple of these pathways could be affected.

Furthermore, complex effects of GS and GT extracts on the pharmacokinetics of different substrates were reported in vitro and in vivo. In the case of GS extract, some in vitro studies showed that GS extract is a potent inhibitor of CYP3A4-mediated metabolism [29]. While other studies showed its ability to induce enzyme expression, as it produced 270 ± 73% of control CYP3A4 mRNA at a concentration of 600 ng/mL in human hepatocytes [84]. Similar in vitro results demonstrated an inhibition of CYP3A4, CYP2C9 and CYP2D6 by GS extract that was standardized to catechin content [85]. In fact, it was shown that the GS extract, when normalized to 1 *μ*M catechin, caused no inhibition to any of the CYP450 isoenzymes’ activity. However, this concentration caused a significant increase in CYP2C9 activity by 30%. When the concentration of GS extract was increased to 10 *μ*M catechin, the activities of CYP2C9 and CYP2D6 was significantly decreased by 58 and 46%, respectively. Furthermore, 10 *μ*M of GS extract almost completely inhibited CYP3A4-catalyzed midazolam 1-hydroxylase activity, and a significant 31% decrease in testosterone 6 *β*-hydroxylase mediated CYP3A4 activity, while increasing the concentration of GS extract to 30 *μ*M of catechin caused similar results to midazolam 1-hydroxylase activity and 86% inhibition of testosterone 6 *β*-hydroxylase activity [85].

GS extract has been shown to inhibit CYP2D6 in vitro [65, 85]. However, a clinical study was conducted to characterize the effect of GS extract on the pharmacokinetics of dextromethorphan, a CYP2D6 substrate, in healthy adult volunteers [86]. It was shown that GS extract caused no significant changes in the metabolic ratio of dextromethorphan and it is safe to be co-administered with drugs that are extensively metabolized by CYP2D6 [86]. Furthermore, one of GS extract’s constituents; resveratrol, showed inhibitory effects against CYP3A4*1 and CYP2D6*1 in vitro in rats and human microsomes, which also was demonstrated in vivo in rats [87]. Resveratrol also inhibited the activities of CYP3A4, CYP2D6, and CYP2C9 enzymes at its pharmacological doses in healthy volunteers study [28].

Interestingly, Nishikawa et al., demonstrated strong inhibition of CYP3A4, CYP2C9, and CYP2D6 activities in human liver microsomes by GS and GT extracts [65]. However, when the effects of a single dose administration of GS and GT extracts were tested in vivo in rats, they showed negligible effects. Nevertheless, increasing the time of both GS and GT extracts’ administration from a single administration to sub-chronic (1 week) administration, it caused an induction of CYP3A in the liver, when midazolam was administered intravenously. This was proven by the increase in hepatic CYP3A protein expression by both extracts. On the other hand, GT extract, but not GS extract, when administered for 1 week, caused a significant increase in both C_max_ and ${\mathrm{AUC}}_0^{\infty }$ of orally administered midazolam, which was suggested to be caused by a reduction in the activity of CYP3A isoenzymes in the small intestines. In fact, intestinal CYP3A protein expression was shown to be decreased after 1 week of GT extract treatment. A suggested explanation of these findings was that GS extracts might act as a selective inducer of the hepatic CYP3A, because of its negligible effects on the intestinal CYP3A. Nishikawa et al. also mentioned that gallate-type catechins, such as epigallocatechin gallate, which is the main component of GT extract that was used in their study, had the lowest penetration through the intestinal walls, despite the fact that it was anticipated to give a strong CYP3A inhibition activity as reported by Muto et al. [66]. Therefore, this suggests that the components of GT extract, which are responsible for CYP isoenzymes inhibition may not be absorbed in the first place. Such in vivo results demonstrated opposite effects of GT extract on CYP3A activity in liver versus in small intestine, which was not shown in vitro [65]. Similar bioavailability issues were also demonstrated for procyanidins (PCs), which are major components of grape seeds [88] and could present as dimers, oligomers, and polymers of the flavan-3-ol monomers (±)-catechin, (−)-epicatechin (EC) and (−)-epicatechin gallate (ECG) [89–91]. Different studies showed that the bioavailability of PCs decreases as the polymerization degree increases, where trimers and larger species have bioavailability that is close to zero and monomers being the most bioavailable [92–95]. Overall, the majority of the ingested PCs remains unabsorbed with relative low bioavailability of 0.3–4% [92–97].

Another controversy was also reported for the modulation of cytochrome isoenzymes activity by GT extract. For example, GT extract caused inhibition of CYP2B6, CYP2C8, CYP2C19, CYP2D6 and CYP3A isoenzymes in human liver microsomes and inhibition of CYP3A in human intestinal microsomes [30]. However, GT extract repeated administration demonstrated no significant effects on CYP 3A4 and 2D6 activities in vivo in healthy volunteers [98]. Nevertheless, GT extract showed increased activity of CYP3A, 1A, and 2B in vivo in rats [99, 100]. On the other hand, GT extract induced the mRNA and protein expression of CYP1A1 and 1A2, while EGCG inhibited CYP3A4 and 1A2 activities in vitro in LS-180 and Caco-2 cell lines [101, 102].

Besides, proanthocyanidins (OPCs) and procyanidin from GS extracts were found to decrease the activity and the expression of multiple ABC transporters in vitro in cancer cell lines [103]. For example, Zhao et al. showed that grape seed procyanidin significantly inhibited P-gp expression by the inhibition of MDR1 gene transcription [104]. Furthermore, resveratrol demonstrated a decrease in the efflux of P-gp substrates and inhibition in ABCB1 mRNA expression in vitro [105]. GT extract’s polyphenols, including catechins such as (−)-epigallocatechin gallate (EGCG), ECG and (−)-catechin gallate (CG) significantly inhibited the activity of P-gp in vitro by increasing the accumulation of rhodamine-123, a P-gp substrate [106]. EGCG also increased the cytotoxicity of another P-gp substrate, vinblastine, in various in vitro models, which indicates that GT extract’s polyphenols, especially EGCG modulate the bioavailability of P-gp substrates at the intestine [106]. In fact, EGCG, (−)-epigallocatechin (EGC), and EC showed 0.1, 13.7, and 31.2% bioavailability, respectively after intragastric administration of green tea in vivo in rats [107]. This could be explained, by EGCG being effluxed by P-gp transporter, which significantly decreased its oral bioavailability [106]. Similar results of multidrug resistance modulation by GT extract and its polyphenols were also reported by other researchers [108–110]. However, (−)-epicatechin (EC) was reported to enhance the P-gp mediated transport of a fluorescent P-gp marker substrate, LDS-751, despite the fact it inhibits the transport of rhodamine 123, in vitro indicating a possibility of EC binding to an allosteric site that increases P-gp activation [111].

Moreover, GT and GS extracts significantly inhibited estrone-3-sulfate uptake mediated by OATP-B by 82.1 and 64.5%, respectively, in human embryonic kidney (HEK) 293 cells [112], which suggests that co-administration of these extracts may decrease the plasma concentrations of OATP-B substrate drug resulting in therapy failure. GT extract inhibition of OATP-B was shown to be concentration-dependent. Furthermore, GT extract constituents; ECG, EGCG and EC, significantly inhibited estrone-3-sulfate uptake by 66.6, 29.5 and 27.2%, respectively, at a concentration of 10 mM. Also, (+)-catechin and EGC significantly inhibited its uptake by 31.7 and 29.1%, respectively, at a concentration of 100 mM [112]. Despite the fact that GS extract consists of higher amount of catechins when compared to GT extract, it was found that their IC_50_ of estrone-3-sulfate uptake for both GS and GT extracts is comparable (~ 22 mg/mL). This may be caused by the differences in the position of flavonoid glycosylation and substituents, for instance, ECG and EGCG (with gallate moiety) showed more potency in the uptake inhibition when compared to (+)-catechin, EGC, and EC (without gallate moiety) [112].

In our study, it is most probably that the resulted effect on the pharmacokinetics of imatinib and *N*-desmethyl imatinib is caused by a combination of multiple kinetic pathways that significantly influence the pharmacokinetics of imatinib and *N*-desmethyl imatinib, other than CYP3A-dependent pathways. For example, the involvement of uptake and efflux transporters’ inhibition in different tissues could be the reason for the shown decreased exposure of imatinib. However, the aforementioned inconsistency between different in vivo and in vitro experiments may be due to the differences in the extracts used and therefore their components [64]. Moreover, the presence of multiple metabolic pathways indicates that the characterization of these pathways requires multiple in vivo models, and a separate in vitro model for the prediction of each metabolite kinetics [113]. The discussed controversies in the literature either between similar models or between in vitro and in vivo models indicate that it is early to propose any mechanism(s) of the characterized co-administration, as the determination of all possible mechanistic molecular and cellular pathways in vitro and in vivo upon co-administration of GS and/or GT extracts with imatinib, are still needed. Furthermore, tissue distribution kinetics are critical and still to be assessed. On the other hand, it is important to remember that in vitro and in vivo animal metabolism data are usually qualitative and not always successful in predicting drug metabolism profiles in humans [114] because of the differences in enzymes’ transcription and regulation [65, 115]. Furthermore, identification of the primary drug metabolites is usually successful using in vitro models [115], but the more complex the drug metabolism, as in imatinib metabolism profile, the more challenging to use in vitro models to characterize in vivo metabolism profiles [114]. Therefore, the relevance of any clinical effects of the characterized interaction requires more investigations and meanwhile caution should be considered in simultaneous administration of GS and/or GT extracts or their components with imatinib.

## Conclusions

The results of this study showed that the co-administration of GS and/or GT extracts and imatinib significantly affects the pharmacokinetics of imatinib and its major metabolite, *N*-desmethyl imatinib. These changes were shown to be affected by the dose administered of each extract with more significant effects when these extracts administered separately rather than mixed. The demonstrated changes in the pharmacokinetics of *N*-desmethyl imatinib are proposed to involve the inhibition of CYP3A-dependent pathway. However, the characterized effects on the imatinib’s pharmacokinetics most probably involve multiple pathways, which include tissue efflux and uptake transporters, in addition to the inhibition of cytochrome isoenzymes. Nevertheless, it is early to determine the dominant affected underlying kinetic pathways, as the characterization of the mechanistic cellular and molecular pathways and tissue distribution of both imatinib and *N*-desmethyl imatinib are still to be assessed. In addition, the significance of the interaction upon co-administration of GS and or GT extracts are yet to be characterized in clinical studies.

## Supplementary Information

**Additional file 1:** **Table S1.** Statistical differences, presented as percentages (%) of decrease or folds of increase, in pharmacokinetic parameters of imatinib among different groups. **Table S2.** Statistical differences, presented as percentages (%) of decrease or folds of increase, in pharmacokinetic parameters of N-desmethyl imatinib among different groups.

## Data Availability

The datasets used and/or analyzed during the current study are available from the corresponding author on reasonable request.

## Abbreviations

ABC: ATP-binding cassette transporters
${\mathrm{AUC}}_0^{\infty }$: Total area under the curve
${\mathrm{AUMC}}_0^{\infty }$: Total area under the first moment curve
BCRP: Breast cancer resistance protein transporter
CG: (−)-catechin gallate
C_max_: The maximum plasma concentration
CYP: Cytochrome P450
DMSO: Dimethyl sulfoxide
EC: (−)-epicatechin
ECG: (−)-epicatechin-3-gallate
EGCG: (−)-epigallocatechin-3-gallate
F: Bioavailability
*h*-GS: High dose of grape seed extract
*h*-GT: High dose of green tea extract
IC_50_: The half maximal inhibitory concentration
IG: Intragastric
GS: Grape seed
GT: Green tea
KTZ: Ketoconazole
LLOQ: Lower limit of quantification
*l*-GS: Low dose of grape seed extract
*l*-GT: Low dose of green tea extract
MRT: The mean residence time
OATP: Organic anion transporting polypeptide
OCT: Organic cation transporters
OPCs: Proanthocyanidin
PC: Procyanidin
P-gp: P-glycoprotein transporter
QC: Quality control
QCH: High quality control
QCL: Low quality control
QCM: Medium quality control
t_max_: Time to maximum plasma concentration
t_0.5_: The terminal half-life
Vz/F: The apparent volume of distribution during the terminal phase
