# Supplementary material for: The effect of grape seed and green tea extracts on the pharmacokinetics of imatinib and its main metabolite, N-desmethyl imatinib, in rats
Source: BMC Pharmacol Toxicol. 2020 Nov 16;21:77. doi: 10.1186/s40360-020-00456-9 (PMC7670682; doi:10.1186/s40360-020-00456-9)
Supplement: Supplementary file 1 — Additional file 1: Table S1. Statistical differences, presented as percentages (%) of decrease or folds of increase, in pharmacokinetic parameters of imatinib among different groups. Table S2. Statistical differences, presented as percentages (%) of decrease or folds of increase, in pharmacokinetic parameters of N-desmethyl imatinib among different groups. [file 40360_2020_456_MOESM1_ESM.docx]

**Table S1:** Statistical differences, presented as percentages (%) of decrease or folds of increase, in pharmacokinetic parameters of **imatinib** among different groups

|  | **Parameter** | | | | | | |
| --- | --- | --- | --- | --- | --- | --- | --- |
|  | **C_max_** | **t_max_** | $\mathbf{t}_{\mathbf{0.5}}$ | $\mathbf{AUC}_{\mathbf{0}}^{\boldsymbol{\infty}}$ | **MRT** | **V_z_/F** | **CL/F** |
| **Imatinib only** |  |  |  |  |  |  |  |
| **Imatinib+ KTZ** | $\downarrow$63.7%* | NS | NS | NS | NS | NS | NS |
| **Imatinib+ *l*-GS** | NS | NS | NS | NS | NS | NS | NS |
| **Imatinib+ *h*-GS** | $\downarrow$61.1%* | NS | $\downarrow$16.1%* | $\downarrow$72.2%* | NS | NS | NS |
| **Imatinib+ *l*-GT** | $\downarrow$53.5%* | NS | $\downarrow$19.6%* | $\downarrow$63.5%* | NS | NS | NS |
| **Imatinib+ *h*-GT** | $\downarrow$49.6%* | NS | $\downarrow$15.8%* | NS | NS | NS | NS |
| **Imatinib+ *l*-GS and *l*-GT** | $\uparrow$ 1.8-fold**^†^**  $\uparrow$ 3.2-fold**^‡^** | NS | $\uparrow$ 1.1-fold**^†^**  $\uparrow$ 1.3-fold**^‡^** | $\uparrow$ 2.6-fold**^†^**  $\uparrow$ 3.9-fold**^‡^** | $\uparrow$ 1.7-fold**^†^** | $\downarrow$59.1%**^†^** | NS |
| **Imatinib+ *h*-GS and *h*-GT** | $\downarrow$60.5%^#^ | NS | $\downarrow$21.9%*  $\downarrow$23.1%^#^ | $\downarrow$66.7%^#^ | $\downarrow$39.8%^#^ | NS | NS |
| *l*-GS and *h*-GS: low and high dose of grape seed extract, respectively. *l*-GT and *h*-GT: low and high dose of green tea extract, respectively. $\uparrow$: Fold of increase. $\downarrow$: Percentage of decrease. ^*^*p*-value < 0.05, compared to control (i.e. imatinib only). ^#^*p*-value < 0.05, when compared to the *l*ow dose of each designated group. **^†^***p*-value < 0.05, when “imatinib+ *l*-GS and *l*-GT” group is compared to *l*-GS group. **^‡^***p*-value < 0.05, when “Imatinib+ *l*-GS and *l*-GT” group is compared to *l*-GT group. | | | | | | | |

Statistical differences, presented as percentages (%) of decrease or folds of increase, in pharmacokinetic parameters of **imatinib**, after imatinib (30 mg/kg) administration alone (control), with ketoconazole (KTZ; 75 mg/kg), with a single low dose of grape seed (GS) or green tea (GT) extracts (*l*-GS or *l*-GT; 50 mg/kg), with a single high dose of GS or GT extracts (*l*-GS or *l*-GT; 100 mg/kg), or with a single co-administered low dose of both GS and GT extracts, or with a single co-administered high dose of both GS and GT extracts. Data: mean ± SD (n=5-6).

**Table S2:** Statistical differences, presented as percentages (%) of decrease or folds of increase, in pharmacokinetic parameters of ***N*-desmethyl imatinib** among different groups

|  | **Parameter** | | | | | | |
| --- | --- | --- | --- | --- | --- | --- | --- |
|  | **C_max_** | **t_max_** | $\mathbf{t}_{\mathbf{0.5}}$ | $\mathbf{AUC}_{\mathbf{0}}^{\boldsymbol{\infty}}$ | **MRT** | **V_z_/F** | **CL/F** |
| **Imatinib only** |  |  |  |  |  |  |  |
| **Imatinib+ KTZ** | $\downarrow$82.8%* | NS | NS | $\downarrow$75.9%* | $\uparrow$ 1.6-fold* | NS | $\uparrow4.3$-fold* |
| **Imatinib+ *l*-GS** | NS | NS | NS | $\downarrow$ 35.1%* | $\downarrow$ $31.2$%* | NS | NS |
| **Imatinib+ *h*-GS** | $\downarrow67.8$%*  $\downarrow63.3$%^#^ | NS | NS | $\downarrow7$3.0%*  $\downarrow58.5$%^#^ | NS | $\uparrow$ 4.1-fold*  $\uparrow$ 2.9-fold^#^ | $\uparrow$ 3.7-fold*  $\uparrow$ 2.5-fold^#^ |
| **Imatinib+ *l*-GT** | $\downarrow$79.3%* | NS | $\uparrow$ 2.6-fold* | $\downarrow$81.1%* | NS | $\uparrow$ 6.8-fold* | $\uparrow$ 5.6-fold* |
| **Imatinib+ *h*-GT** | $\downarrow$63.3%* | NS | $\uparrow$ 1.2-fold* | $\downarrow$64.3%* | NS | NS | $\uparrow$ 4.3-fold* |
| **Imatinib+ *l*-GS and *l*-GT** | $\uparrow$ 4.9-fold**^‡^** | NS | $\uparrow$ 1.3-fold**^†^** | $\uparrow$ 1.8-fold**^†^**  $\uparrow$ 6.1-fold**^‡^** | $\uparrow$ 1.6-fold**^†^** | NS | NS |
| **Imatinib+ *h*-GS and *h*-GT** | $\downarrow$71.5%*  $\downarrow$71.9%^#^ | NS | $\uparrow1.6$-fold*  $\uparrow1.3$-fold^#^  $\uparrow1.5$-fold^$^  $\uparrow1.4$-fold^§^ | $\downarrow$72.1%*  $\downarrow$75.8%^#^ | NS | $\uparrow$8.7-fold*  $\uparrow$8.8-fold^#^ | $\uparrow$5.3-fold*  $\uparrow$6.5-fold^#^ |
| *l*-GS and *h*-GS: low and high dose of grape seed extract, respectively. *l*-GT and *h*-GT: low and high dose of green tea extract, respectively. $\uparrow$: Fold of increase. $\downarrow$: Percentage of decrease. ^*^*p*-value < 0.05, compared to control (i.e. imatinib only). ^#^*p*-value < 0.05, when compared to *l*ow dose of each designated group. **^†^***p*-value < 0.05, when “imatinib+ *l*-GS and *l-*GT” group is compared to *l*-GS group. **^‡^***p*-value < 0.05, when “imatinib+ *l*-GS and *l-*GT” group is compared to *l*-GT group. ^$^*p*-value < 0.05, when “imatinib+ *h*-GS and *h*-GT” group is compared to *h*-GS group. ^§^*p*-value < 0.05, when “imatinib+ *h*-GS and *h*-GT” group is compared to *h*-GT group. | | | | | | | |

Statistical differences, presented as percentages (%) of decrease or folds of increase, in pharmacokinetic parameters of ***N*-desmethyl imatinib**, after imatinib (30 mg/kg) administration alone (control), with ketoconazole (KTZ; 75 mg/kg), with a single low dose of grape seed (GS) or green tea (GT) extracts (*l*-GS or *l*-GT; 50 mg/kg), with a single high dose of GS or GT extracts (*l*-GS or *l*-GT; 100 mg/kg), or with a single co-administered low dose of both GS and GT extracts, or with a single co-administered high dose of both GS and GT extracts. Data: mean ± SD (n=4-6).
